# Testing effects of trigeminal stimulation on binary odor mixture quality in rats

**DOI:** 10.3389/fnins.2023.1059741

**Published:** 2023-03-07

**Authors:** Huibo Li, Clara Lee, Leslie M. Kay

**Affiliations:** ^1^Department of Psychology, The University of Chicago, Chicago, IL, United States; ^2^Institute for Mind and Biology, The University of Chicago, Chicago, IL, United States; ^3^The College, The University of Chicago, Chicago, IL, United States

**Keywords:** odor mixture, trigeminal, overshadowing, two-alternative choice, partial reinforcement, binary mixture perception

## Abstract

Prior attempts at forming theoretical predictions regarding the quality of binary odor mixtures have failed to find any consistent predictor for overshadowing of one component in a binary mixture by the other. We test here the hypothesis that trigeminality contributes to overshadowing effects in binary mixture perception. Most odorants stimulate the trigeminal nerve in the nasal sensory epithelium. In the current study we test rats’ ability to detect component odorants in four binary odor sets chosen for their relative trigeminality. We predicted that the difference in trigeminal intensity would predict the degree of overshadowing by boosting or suppressing perceptual intensity of these odorants during learning or during mixture perception. We used a two-alternative choice (TAC) task in which rats were trained to recognize the two components of each mixture and tested on a range of mixtures of the two without reinforcement. We found that even though odorant concentrations were adjusted to balance volatility, all odor sets produced asymmetric psychometric curves. Odor pairs with the greatest difference in trigeminality showed overshadowing by the odorant with weaker trigeminal properties. Odor sets with more evenly matched trigeminal properties also showed asymmetry that was not predicted by either small differences in volatility or trigeminality. Thus, trigeminal properties may influence overshadowing in odor mixtures, but other factors are also likely involved. These mixed results further support the need to test each odor mixture to determine its odor quality and underscore recent results at the level of olfactory receptor neurons that show massive and unpredictable inhibition among odorants in complex mixtures.

## 1. Introduction

The ability to discriminate and identify odors plays an important role for many animal species, because they rely on the sense of smell to eat, mate and detect danger. Most natural odors are compounds of large numbers of molecules. For instance, a natural floral scent could contain hundreds of components (Levin et al., 2003). Despite the complexity of natural odors, most olfactory research in rodents has relied on monomolecular odorants or has used mixtures assuming that equal concentrations result in equal perceptibility. Understanding the quality of mixtures appears a simple problem at first—examine the receptor or glomerular input patterns and add them together in the way that one might for different colors of light that combine to form a visual percept. However, predicting what a mixture of even just two odorants will smell like remains a difficult task (Kay et al., 2005; Frederick et al., 2009). While similarity in quality between complex mixtures can be predicted to some degree in humans (Ravia et al., 2020), we still do not understand the physiological mechanism for this process in rats or humans.

The factors that lead to the complexity of odor mixtures often stem from peripheral effects. Odorant sorptiveness can affect the detectability of components in a binary mixture (Rojas-Líbano and Kay, 2012). Glomerular activation patterns from mixtures can be quite different from their components (Grossman et al., 2008). Early work hinted at non-linear effects due to inhibition in mixtures at receptors (Araneda et al., 2000), and knowing about these interactions can help predict some aspects of odor quality (Kay et al., 2003). Recent work from the Firestein lab shows massive inhibitory interactions occur among components of odor blends at the level of the odor receptors on the olfactory sensory neurons (Xu et al., 2020). This inhibition may be the force behind the subtlety of perfumery and odor accord profiles.

The trigeminal profile of component odorants may also affect mixture quality. Trigeminality or chemesthesis is responsible for the sensations of hot, cold and irritation from substances like chiles, mint and CO_2_, and these sensations are supported by many types of Transient Receptor Potential (TRP) cation channels on the trigeminal nerve. Research on TRP channels and their role in trigeminal sensations has provided evidence for the integral role the trigeminal system plays in olfactory experience (Silver et al., 2006; Hansen and Finger, 2008; Baxter et al., 2021).

Most odorants have trigeminal properties, and stimulation of the trigeminal nerve in the nasal epithelium can influence odor detection and recognition (Hummel and Livermore, 2002; Jacquot et al., 2004, 2010), sensitivity (Buron et al., 2009; Frasnelli et al., 2011; Galliot et al., 2012), perceptual memorability (Han et al., 2018), and intensity (Cain and Murphy, 1980; Kobal and Hummel, 1988; Livermore et al., 1992).

There is a direct neuromodulatory route from the trigeminal ganglion to the olfactory bulb (OB) (Schaefer et al., 2002). Trigeminal nerve endings release CGRP and substance P in the nasal epithelium when stimulated, and some collaterals of the trigeminal nerve enter the OB with the olfactory nerve, where the same neuromodulators can affect OB neuron firing in response to odors (Finger and Bottger, 1993; Schaefer et al., 2002; Stanić et al., 2010; Genovese et al., 2017; Messlinger et al., 2020). Trigeminal stimulation has been shown to influence the intensity of odorants both positively and negatively in humans (Cain and Murphy, 1980; Kobal and Hummel, 1988; Livermore et al., 1992).

All of these factors suggest a role for trigeminal stimulation in the perceptual quality of mixtures. The perceptual quality of binary mixtures can be categorized as elemental (component odors are recognized), configural (synthetic percepts in which the components are not recognized), and overshadowing (the mixture smells like one of the odorants) (Linster and Smith, 1999; Kay et al., 2003, 2005; Wiltrout et al., 2003; Coureaud et al., 2009, 2020). We hypothesize here that because trigeminal stimulation can affect detection of odorants, it could play a significant role in binary odor mixture perception. Specifically, we expect that trigeminal activation can contribute to overshadowing in binary mixtures. Our paradigm is designed to examine detectability of individual odorants in mixtures. Trigeminal activation can make odors more or less detectable. Thus, we expected that strong trigeminal stimulation would boost or suppress detection or learning of individual odors, contributing to differences in detectability of the components of a binary mixture. This would show up perceptually as overshadowing.

Based on limited knowledge of the trigeminal profiles of a few odorants, we test our hypothesis by controlling the trigeminal difference in the two component odorants in binary mixtures. We expect an odor mixture with component odorants of greater trigeminal difference to show greater overshadowing of one component by the other, or greater differences in detectability of the two odorants.

We tested the overshadowing effects of binary odor mixtures at varying combination ratios to determine the degree of overshadowing. We trained rats to respond to the two pure mixture components and then show us in a partial reinforcement two-alternative choice (TAC) paradigm whether various mixture ratios smell more like one or the other component. We expected binary odor mixture psychophysical curves to have inflection points which depend on the difference in trigeminality instead of symmetric curves with inflection points at equal vapor phase concentration of the two odorants.

We further expected that the more different the trigeminal intensities of the components, the further away the point at which the two components are equally recognizable (inflection point) would be from equal concentrations of the two odorants. The direction of shift was predicted to be either toward the more or less trigeminal odorant.

## 2. Materials and methods

### 2.1. Subjects

Adult Sprague Dawley rats were purchased from Envigo, Indianapolis, IN, USA and began training at 8–10 weeks of age. We began with six males and six females, but one male and one female were excluded for failure to learn the task. We did not track estrus stage of the female rats, because female rats and mice do not produce more variable results than males across many measures (Prendergast et al., 2014; Becker et al., 2016), and we assumed that testing over the many weeks would randomize any rhythmicity in female rats’ variability. Furthermore, testing of the ten rats was not synchronous, smaller subgroups were trained and tested in sequence, which would further smooth out any possible rhythmicity. Therefore, all rats were trained and tested in the same way regardless of sex.

Rats were dieted to 85% of their *ad libitum* weight before beginning training and were maintained on a restricted food schedule for the duration of the experiment. All rats were housed singly in standard housing cages with filter tops and maintained on a 14/10 h light/dark cycle (lights on at 8:00 a.m. CST). All experiments were performed during the light phase between 9 a.m. and 6 p.m. CST to avoid exposing rats to light during the dark phase (Travlos et al., 2001; Bedrosian et al., 2013). To avoid variance within subjects across days due to circadian effects and to enable behavioral entrainment to the rewards given at the time of testing, each rat was tested in a set time window on each testing day (Carneiro and Araujo, 2012). All experimental procedures were done under veterinary supervision with approval and oversight by the University of Chicago Institutional Animal Care and Use Committee, according to the Association for Assessment and Accreditation of Laboratory Animal Care guidelines.

### 2.2. Apparatus

All experiments were conducted in two identical operant chambers with identical odor delivery systems. Operant chambers (ENV-008, Med Associates, Georgia, VT, USA) and odor delivery systems were modified based on established protocols in our lab (Frederick et al., 2011), to be able to deliver seven combinations of binary mixtures (Solenoid manifolds were NR Research 225T092, and flow meters were MasterFlex MFLX32003 series). Saturated vapor was maintained by bubbling clean air through columns of pure liquid odorants. To control for differences in volatility between the odorants in an odor set, flow rates of each component odorant in each odor set were adjusted in inverse ratio to the ratio of the theoretical vapor pressures of the two odorants. For mixtures, separate lines for each odorant were combined and then combined with plain air. See Table 1 and Section “2.4 Testing” for details on ratios. All odorized air was combined with a diluting clean air stream of 1 LPM. The odor lines were charged for 1 s before the rats could trigger odor delivery, with odorant removed by the vacuum line just before the odor port until the nosepoke occurred. Timing of event inputs and outputs were controlled and logged by a computer running MedAssociates MedPC IV software.

**TABLE 1:** Odor set details.

	Odorant A	VP @ 25C (Pa)	TRP	100% flow (LPM)	Odorant B	VP @ 25C (Pa)	TRP	100% flow (LPM)
OS0 (Training)	Amyl acetate	650	–	0.15	Anisole	472	–	0.15
OS00 (Transition)	Butyl acetate	1533	–	0.15	Hexanol	124	–	0.15
OS1	Eugenol	2.95	V1, V3, A1, M8	0.2	PEA	11.57	–	0.05
OS2	Eucalyptol	253.31	A1, M8	0.16	+Limonene	205.5	A1	0.2
OS3	Eugenol	2.95	V1, V3, A1, M8	0.2	Cinnam-aldehyde	3.85	A1	0.16
OS4	Citral	12.17	V3	0.19	PEA	11.57	–	0.2

A total of 100% flows of component odors are controlled to be approximately in inverse ratio of theoretical vapor pressures. OS1 and OS4 are composed of components with higher trigeminal difference. TRP channel subtypes that are activated by each component of OS1- 4 are indicated, where known. All 100% flows and odor mixtures were then combined with 1 LPM of clean air.

### 2.3. Two-alternative choice paradigm–training

All rats were trained to perform a TAC behavior once they reached their respective 85% *ad libitum* weights. The TAC protocol trains rats to associate each of two different monomolecular odorants (saturated vapor from a single odorant mixed into the plain air stream) with one of two nose poke ports (left/right) in three phases. Rats were trained to associate odor A with the left port and odor B with the right port for all odor sets. Previous experiments have shown that in this TAC task, rats do not maintain a side bias after training, and therefore we did not balance the side of the two odors across subjects (Frederick et al., 2011, 2017).

#### 2.3.1. Phase 1

Rats were trained to poke their nose into the odor port on the center of the front aluminum wall upon house light signal onset. Every time rats poked into the odor port, one 45 mg sugar pellet was dispensed as a reward (Bioserv 45 mg Dustless Precision Pellets). The odor port delivered odorants when rats had their nose inside the port, which was detected by an infrared (IR) detector situated at the edge of the odor port. For phases 1 and 2, the odor was amyl acetate. A vacuum line was always on, removing any odor from the supply line just before the odor port, unless the IR beam was disrupted. Thus, odor delivery, triggered by IR beam interruption during the nose poke, had a very short delay, on the order of a few tens of milliseconds. The odor stayed on as long as the beam was disrupted. Only one odor sampling period was allowed during each trial. Rats successfully learned phase 1 (50 correct trials) in 2.3 ± 0.5 days.

#### 2.3.2. Phase 2

Rats were trained to associate Odor A with the left response port. Every time rats sampled from the center odor port and then nose poked at the left response port within 5 s, one sugar pellet was dispensed as a reward. Rats reach above 95% accuracy in 2.6 ± 1.3 days.

#### 2.3.3. Phase 3A

Rats were trained to associate Odor B with the right response port. During training, odor B was anisole. Odor A or B was randomly chosen on each trial. Rats received a reward for all correct responses (left port Odor A and right port Odor B) made within 5 s after nose withdrawal from the odor port. Rats trained on Phase 3A (8.7 ± 3.4 days) until they performed at 70% accuracy or better for two consecutive days.

#### 2.3.4. Phase 3B

Rats were trained to perform the learned association with partial reward, Starting at 90% reinforcement and dropping to 60% in 10% decrements. Each rat was trained at 100% reinforcement for each odor pair and then reduced to 60% reward probability. By the end of phase 3B, all rats included completed 300 attempts in one session on each training day and performed at over 70% accuracy with 60% reward probability for two consecutive days.

### 2.4. Testing

Rats performed the above training with two sets of training odors before being tested on treatment odor sets. The first training set was for task learning; the second training set was to avoid transition (rule transfer) effects (Frederick et al., 2017). Training of the first training odor set (OS0) was composed of Phases 1, 2, and 3 described above. Training of all other odor sets began with phase 3. Only treatment odor sets were used in Phase 4, the testing phase.

#### 2.4.1. Phase 4

Rats performed the same odor discrimination task they had learned, not only with odorants A and odorant B, but also with binary mixtures of odorants A and B. On each trial, either a pure odorant was chosen randomly between odorant A (100A–0% B) and odorant B (0A–100% B), or a binary mixture at one of five ratios was selected. The five ratios were 75A–25% B, 55A–45% B, 50A–50% B, 45A–55% B, 25A–75% B (the percent symbol for combination ratios are omitted in later passages for convenience). Percentages reflect partial flows of the respective pure odorants’ saturated vapor.

Flow rates were adjusted to account for the relative theoretical vapor pressures (volatilities) of the two odorants, and all odorants were then mixed with plain air. Pure odorant A and pure odorant B flows were calculated so that the ratio of flows equals the inverse ratio of their respective theoretical vapor pressures at 25°C. In other words, theoretical intensities of 100A and 100% B are controlled to be the same. A binary mixture composed of 50% flow of odorant A’s pure odorant and 50% flow of odorant B’s pure odorant thus has, theoretically, two equal components in terms of vapor phase concentration. In the binary mixture, when the percentage of odorant A is higher, it reflects that the binary mixture is composed of more A than B molecules. The odor flows (ranging from 0.01 to 0.2 LPM) were then mixed with plain air at 1 LPM.

The number of trials was controlled so that there were 300 total trials, composed of 200 pure A or B trials (evenly divided between odorant A and odorant B) and 100 mixture trials (evenly divided between the five kinds of mixture types) with the order of all trial types randomized. The reward probability for monomolecular odorant (odorant A or B only) correct trials was 80%, and the reward probability for all mixture trials was 20%, regardless of which odor port was chosen. This enabled the overall reward probability to be kept at 60% without biasing rats’ responses to mixture trials due to learning a reward influence. For each trial, the rat’s response (left or right port) was recorded. If a response to a pure odorant (100% A or B) trial was incorrect, the normal 7 s penalty delay was imposed. There was no penalty for not responding and no penalties on mixture trials.

Phase 4 spanned 2 days for each odor set. During these two consecutive days, rats performed the session described above. A session ended when rats completed 300 trials. All rats were able to meet this criterion for at least two odor sets not including training and transition odor sets. Due to shutdown of the lab at the beginning of the pandemic, long delays between odor sets resulted in failure of some rats to reach criterion performance on some of the odor sets. The final number of rats that completed odor sets 1–4, respectively, is 6, 10, 7, 8.

### 2.5. Odors

Research with humans who are anosmic provides a psychological measure of trigeminal intensity for some odorants. Combining such information with the list of agonists and antagonists of the relatively well-studied TRP channel families, we selected four sets of odorants as the component odorants of binary mixtures. Odorants are matched based on their estimated trigeminal profiles (from human data) and volatilities. Table 1 displays all odor set pairing details.

Of the four odor sets (OS), OS1 (eugenol/PEA), and OS4 (citral/PEA) have the best documented large trigeminal difference (Doty et al., 1978; Lübbert et al., 2013). PEA is the only known odorant that is liquid at room temperature and very low in trigeminal intensity. OS3 (eugenol/cinnamaldehyde) has components that have relatively similar trigeminal profiles; their TRP channel activation profiles overlap, and both have been rated as trigeminal odors in human anosmia research (Doty et al., 1978; Bandell et al., 2004; Kollndorfer et al., 2015). OS2 [eucalyptol/(+)limonene] has components that have possibly close trigeminal intensities; however, limonene is likely to be of lower trigeminal intensity than eucalyptol according to TRP channel activation profiles (Nilius and Owsianik, 2011; Takaishi et al., 2012; Kaimoto et al., 2016; Chandorkar et al., 2021) and human research data (Doty et al., 1978; Kobal and Hummel, 1988). Despite limited data on limonene trigeminal intensity in animal research, given the similarity between the human and rat olfactory systems, we use human data as a guideline. The vendor and CAS numbers for all odorants used are: PEA (Sigma-Aldrich, 60-12-8), eugenol (Sigma-Aldrich, 97-53-0), citral (Fluka, 5392-40-5), cinnamaldehyde (Sigma-Aldrich, 14371-10-9), (+)limonene (Sigma-Aldrich, 5989-27-5), eucalyptol (Sigma-Aldrich, 470-82-6).

### 2.6. Analysis

Analysis was guided by two goals: (1) characterization of individual odorant recognizability in binary mixtures when component ratio is equalized in the vapor phase (the 50–50 mixtures), and (2) investigation of the effect of trigeminal difference on response frequencies to component odorants of the binary odor mixture. Both of these goals are focused on understanding deviations from symmetry of a psychometric curve around the 50–50 point.

Based on our design, there are four independent variables in the analysis: combination ratios, odor set, sex, and testing day. There are two dependent variables, response and sampling duration. For each trial, sampling duration, response delay, and response side (left/A or right/B) were recorded. Sampling durations and responses are calculated as below.

Response: for each trial, the odor combination ratio and the port that the rat responded to were recorded. The binary values (left or right) for each combination ratio were then calculated into a continuous session parameter with possible values ranging from 0 to 1. The final dependent variable response intensity for each combination ratio is calculated as (n go left/N total) for each odor combination ratio. While the code was written to exclude trials in which rats did not respond, there were no such trials in the data set.

Sampling Duration: the amount of time that the rat had its nose in the odor port (time between nose entry and exit). Although rats were free to nose-poke again, the odorant was on only for the duration of the first nose-poke. Therefore, we used only the time for which the animal could receive odorant.

Univariate and multivariate ANOVAs were conducted in R using the function *aov*. For analysis of response, interaction terms were selected with a focus on combination ratio, because we expected combination ratio to be a significant factor. Hence, to check for latent learning effects based on combination ratios due to 2 days of testing, we included the interaction between combination ratio and test day in the model. To determine whether different mixtures produced different response patterns across the combination ratios, we included interaction between combination ratio and odor set.

Bootstrapping was conducted using a custom function based on the R functions *slice_sample* and *replicate*. Grouping was based on rat, odor set, and combination ratio. Within each group, sampling (*n* = 36, to match approximately the number of our data points) was done with replacement, with 1,001 iterations. Bimodality tests were conducted with R using the function *bimodality_coefficient* in the mousetrap package (Wulff et al., 2021). Logistic regression was conducted with the R *glm* function. T-tests were conducted with the R function *t_test*.

Cohen’s d and partial eta-square (η^2^_*p*_) statistics were used to evaluate effect sizes. Cohen’s d was calculated based on pilot data to determine effect size and subject numbers. Using the mean response intensity from six rats on OS1, we simulated data with group mean response intensity as 0.5 at 50A–50B. The standard deviation for the simulated no-effect outcome was assumed to be the same as that of the pilot data. Six subjects produced a large effect size (Cohen’s *d* = 2.1805436), so our 10 subjects are a large enough sample to evaluate our hypothesis. Partial eta squared was calculated with R using the function *partial_eta_squared* based on ANOVA results.

Unless otherwise stated within the text, all analysis was conducted with an alpha value of 0.05. Analyses were done in MATLAB (vR2017a) and R (version 4.0.3). Statistical results are reported in the text.

## 3. Results

We hypothesized that the relative trigeminal strengths of the two odorants in each mixture would have differing effects on the perceptual quality of odorants. To test this hypothesis, we trained rats in a TAC task to indicate whether the binary mixture smelled more like one component or the other. We tested rats across four odor sets chosen for their trigeminal differences. There was no difference in phase 3 learning rate across the four test odor sets (*p* = 0.48). Rats learned all odor sets in 3.5 ± 1.1 days.

### 3.1. Perceptual qualities of binary mixtures vary in different manners across combination ratios

We used the Left/Right response ratios for each mixture to determine whether there were significant differences across mixture ratios and odor sets in addition to the influence of sex and test day. We performed a repeated measures multi-way ANOVA on the dependent variable response (proportion of left side responses, where 1 = left 100% of the time and 0 = right 100% of the time) and the independent variables combination ratio, test day, sex, and odor set. We also tested for interactions between combination ratio and test day, and combination ratio and odor set, assuming there would be differences in how rats respond to ratios that depend on either the odor set or on slow learning of the mixtures. We found a significant main effect of combination ratio on response [*F*_(1,440)_ = 312, *p* < 0.001] with a large effect size (η^2^_*p*_ = 0.41). We also found a significant main effect of odor set on response [*F*_(1,440)_ = 11.26, *p* < 0.001]. Both combination ratio and odor set influenced perceptual qualities of the binary odor mixtures, meaning whether the rats responded more to one component or the other depended on the ratio and the odor set. No other variables, test day, sex, interaction between combination ratio and test day, or interaction between combination ratio and odor set showed significant effects. Of the two interaction terms included, the interaction between combination ratio and odor set, contrary to our expectations, does not significantly predict response. It is possible that given the logistic nature of response on all odor sets, the multi-way ANOVA does not offer the sensitivity required to capture the fine differences in response side on the combination ratios around 50A–50B. The main effect of odor set supports our hypothesis that different binary mixtures have different characteristic psychometric curves. The following analysis for response collapses data across test days and sex.

**FIGURE 1:**
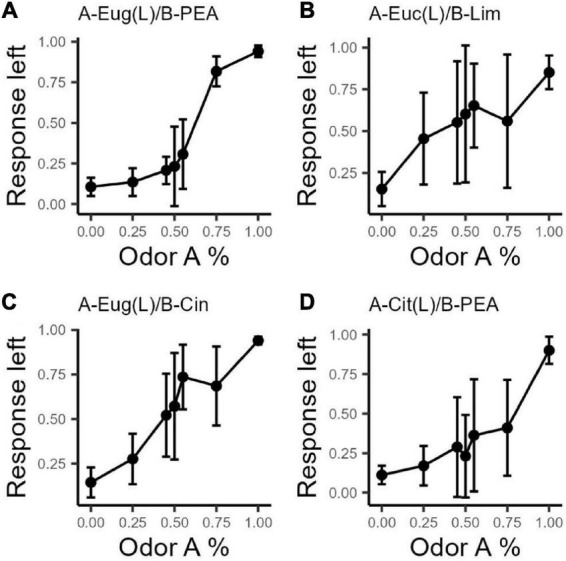
Response intensity to the left port of each odor set. Response intensity is calculated as (n go left/N total) for each odor combination ratio (response left on vertical axis). Odorant A corresponds to the left port. Error bars are based on the standard deviation across subjects for each odor concentration ratio (horizontal axis). All odor sets show a logistic trend. Variances at extreme combination ratios (0A and 100A) are smaller than those of other combination ratios for all odor sets. **(A)** *n* = 6, OS1: Eug/PEA; **(B)** *n* = 10, OS2: Euc/Lim; **(C)** *n* = 7, OS3: Eug/Cin; **(D)** *n* = 8, OS4: Cit/PEA.

Figures 1A–D shows the mean responses to the left (odor A) operant port for all odor sets plotted against the percentage of odor A in each mixture A and B. For each odor set, the mean go-left frequency for all rats that finished the odor set (*n* = 6, 10, 7, 8, for the 4 odor sets), at seven odor combination ratios, is calculated across days and sessions. Variances for combination ratios around 1:1 (45A–55B, 50A–50B, 55A–45B) are higher than at the extremes (0A–100B and 100A–0B) for all odor sets, consistent with the expectation that when the odorants are in equal measure in the mixture, the perceptual quality is more ambiguous and the response more variable.

Variance within an odor set was large in some cases. The variance for the Eugenol/PEA odor set appeared smaller than that of the other three odor sets. Based on the raw data distributions (Supplementary Figure 1), we noted that within some odor sets, rats showed apparent subgroups in response patterns. For instance, odor set 2, Eucalyptol/Limonene shows two possible groups. Therefore, we conducted further variance analysis to determine whether there were subgroups in responding within odor sets.

**FIGURE 2:**
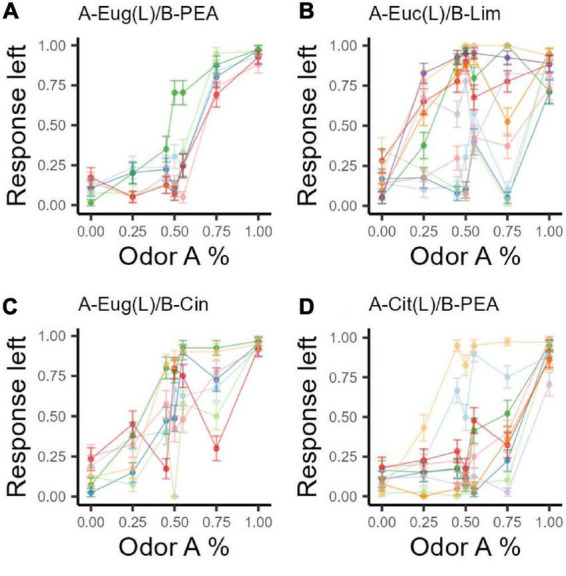
Bootstrapped response intensity to the left port for each odor set. Based on the original binary response, grouping by odor set, rat, and combination ratio, we sampled with replacement (36 times to match number of data points in our original data) data points over 1,001 iterations. The response ratio to the left port of each rat on each combination ratio for each odor set is again calculated as (n go left/N total) for each odor combination ratio. Error bars are standard deviations. Colors indicate rat identities; each rat has the same color across panels. **(A)** *n* = 6, OS1: Eug/PEA; **(B)** *n* = 10, OS2: Euc/Lim, OS2 shows two clear subgroups, one group goes to the left port more than 50% of the time at 50A while the other group goes to the right; **(C)** *n* = 7, OS3: Eug/Cin; **(D)** *n* = 8, OS4: Cit/PEA.

For each combination ratio, we generated bootstrapped responses from all subjects. We then computed a bimodality coefficient (BC) on the bootstrapped responses for each odor mixture ratio within each odor set using functions in the R package, Mousetrap, to calculate the BCs (Pfister et al., 2013). Our criteria for significant subgroups were based on the BCs value and the number of high BC values in consecutive order in each odor set. The criterion for the distribution of response on each combination ratio to be significantly bimodal is a BC > 0.555. To determine whether the distributions of overall responses were bimodal, we required the BCs of at least four adjacent combination ratios within that odor set to be >0.555. Furthermore, we required that the bimodality be driven primarily by the same subjects within each mixture ratio. See Figures 2A–D for point by point (combination ratio) mean response to left for each rat (indicated by color identity) based on bootstrapped data. The bimodal distribution can thus be interpreted as the sum of two normal distributions, each representing a group of rats that responded similarly. Using these criteria, we concluded that the response distributions for odor set 2 (Eucalyptol/Limonene) and odor set 4 (Citral/PEA) each have two subgroups of subjects (see details in Supplementary Table 1).

### 3.2. Asymmetry of psychometric curves

**FIGURE 3:**
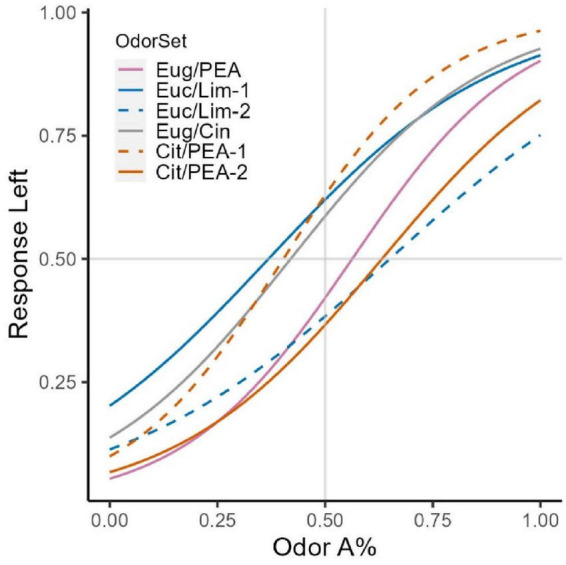
Psychometric curve fits for all odor sets. A logistic model was fit to each odor set’s response intensities. Subgroups identified for OS2 and OS4 based on bimodality tests are treated as separate groups. For those groups, solid curves indicate the subgroup with more subject numbers (OS2 larger group: *n* = 6, smaller group *n* = 4; OS4 larger group *n* = 6, smaller group *n* = 2). Larger differences in trigeminal profiles do not produce larger overshadowing effects. OS1 (Eug/PEA) and OS4 (Cit/PEA) do not produce more shifted curves.

For each odor set, we fit the response to the left to a logistic general linear model with the percentage of odor A as the predictor. Odor sets that passed the bimodality tests were analyzed by subgroup. For all models, the percentage of odor A in the mixture significantly predicts rats’ go response (all *p* < 0.001). Figure 3 shows the predicted response frequency to the left for all percentages of odor A in mixtures. None of the odor sets have a predicted value of 0.5 for response frequency to the left when the composition of components of the binary mixture is 1:1. This argues against the idea that binary mixtures have uniform perceptual qualities across the spectrum of possible combination ratios. Moreover, around component ratio 1:1, different odor sets behave differently. All odor sets falsify the hypothesis that the recognizability of component odorants in a binary mixture is symmetrical about the 50–50 concentration ratio. It appears that each binary odor set is unique.

There are two ways to test the hypothesis that OS1 and OS4, the odor sets with components of high trigeminal difference, show a more dramatic overshadowing effect. The first way to look at the fitted curves is that, when chemical concentration is controlled to be 1:1 (combination ratio = 50A–50B), response intensity to the left port should not equal 0.5. If response intensity to the left port is lower than 0.5, it means rats go to the right port more when chemical intensity is the same for both components, or odor B (the less trigeminal odorant) overshadows odor A. If response is higher than 0.5, this means that rats go to the left response port more than the right one at the 50–50 ratio, or odor A (the more trigeminal odorant) overshadows odor B. The second way to look at the fitted models is when response intensity is equal to 0.5 (i.e., when rats report the mixture to smell like either component odor equally), the corresponding mixture ratio should not be 1:1 (50A–50B) if either odor overshadows the other. If the point at which the response intensity to the left port equals 0.5 is to the left of 50A–50B, A overshadows B. If the point at which the response intensity to the left port equals 0.5 is to the right of 50A–50B, then B overshadows A.

OS1 and OS4, with PEA as odorant B, have the best documented high trigeminal difference between components (Doty et al., 1978; Lübbert et al., 2013). OS1 does not show a larger shift of inflection point away from 50 to 50 than do OS2 and OS3. It is therefore not true that components with larger trigeminal difference show a more dramatic overshadowing effect. However, both OS1 and OS4 curves show that PEA, the non-trigeminal odorant, overshadows the trigeminal odorant.

### 3.3. Sex and odor set differences in sampling duration

Longer sampling duration has been linked to better performance in odor discrimination (Frederick et al., 2017). The distribution of sampling durations within a session is skewed, so the median is a better measure of within-session behavior. Across sessions, the distribution of medians is normal (Frederick et al., 2011). To check the influence of different trial types, or various combination ratios, on rats’ decision-making strategies, we performed repeated measures ANOVA on the independent variable median sampling duration and dependent variables test day, combination ratio, sex, and odor set. Combination ratio was not a significant predictor of sampling duration [*F*_(1,442)_ = 1.5, *p* = 0.22]. This supports the effectiveness of our protocol in testing rats’ responses to binary mixtures of various ratios. Rats do not employ different sampling strategies in responding to mixtures and pure odorants, meaning that, given the same sampling time across mixtures, the odor quality is well-represented by the response.

We found sex [*F*_(1,442)_ = 3.88, *p* = 0.0495] and odor set [*F*_(1,442)_ = 100.99, *p* < 0.001] to be significant factors in predicting sampling duration. *Post-hoc t*-tests with Bonferroni’s correction show that only the OS4 average median sampling durations are significantly different between male and female rats. This significance is most likely driven by 1 male rat who consistently sampled longer than 1 s for OS4. The mean sampling durations for all rats are shown in Supplementary Figure 2. Similar to previous studies (Frederick et al., 2011, 2017), there is high variance in sampling duration across rats and odor sets. Other studies addressing sex differences in olfaction, including work done in our lab, indicate that male and female rats show differences in sampling time in non-learning tasks (Perez et al., 2018; VM et al., 2022). In our current study, female rats show a trend to sample longer than males for OS1, but shorter for OS2-4.

## 4. Discussion

The majority of binary mixtures are not elemental in perceptual quality, and most show some type of overshadowing effect by one of the odorants over the other (Wiltrout et al., 2003; Kay et al., 2005; Frederick et al., 2009). However, we do not know what gives rise to perceptual overshadowing or how to predict the predominance of one odor in a binary mixture. Our hypothesis rested on the expectation that trigeminal profiles of the components of a binary mixture would reveal information on the direction and size of overshadowing. We recorded rats’ responses to mixtures of different ratios to examine the relationship between trigeminal difference and overshadowing in binary odor mixture quality. We used four odor sets of varying trigeminal profiles to test the hypothesis that larger trigeminal differences between the two odorants in the binary mixture would predict more overshadowing.

We found that 50–50 mixtures of binary odors did not coincide with equal perceptibility of the two component odorants for all of the odor sets tested (Figure 3). For both odor sets with large trigeminal differences, the non-trigeminal odorant (PEA) overshadowed the strong trigeminal odorant (eugenol or citral), except for the small subgroup of two rats in the citral/PEA test (Figure 3). However, trigeminal differences between components did not predict the magnitude of overshadowing effects. These results support our hypothesis that trigeminal activation may drive the directional properties of overshadowing. Our data suggest that the less trigeminal odorant overshadows the more trigeminal one, which could be due to a decrease in perceptual strength of the trigeminal odorant during learning. A caveat is that there are very few known low or non-trigeminal odorants that are liquid and volatile at room temperature, and the only odorant in that category that we tested was PEA. This could be a specific effect of PEA in the binary mixtures we tested.

It is likely that overshadowing dynamics involve a multitude of factors including chemical structure, volatility, sorptiveness, trigeminality, mixture ratio, agonistic and antagonistic interactions with olfactory receptors, and more. To understand overshadowing dynamics, we would benefit from systematizing the trigeminal profiles of commonly used volatile chemicals. Given the progress made in understanding TRP channels, which give rise to chemesthetic properties, there is much work left to be done to characterize the trigeminal profiles and intensities of commonly used odorants. In selecting more trigeminal odorants, we assumed that chemicals that activate different families of TRP channels elicit generalizable levels of intensities. Future studies comparing the relative intensities of chemicals that activate different subfamilies of TRP channels, for example those that active TRPV1 and TRPM8 when concentrations are controlled to be the inverse ratio of respective vapor pressure, will enable us to characterize perceptual qualities of both monomolecular and mixture odorants more strictly.

None of the mixtures showed a symmetric sigmoid relationship of component perceptibility. We do not yet understand completely what drives the asymmetry, and there appear to be individual differences as well, as exemplified by the subgroups of subjects’ response patterns within two of the odor sets. We note that some studies of odor psychophysics and electrophysiology assume that psychophysical curves depend on the liquid or gas phase concentration of odorants, independent of their physical properties or possible interactions at the receptor level either directly through inhibition or indirectly *via* trigeminal nerve activation (e.g., Uchida and Mainen, 2003). Because all the psychophysical curves we found were asymmetric, our results suggest that training subjects to treat ratios of binary mixtures as perceptually symmetric may add unplanned and unpredictable cognitive load to the animals performing the task. The physiological interpretations or behavioral strategies can then be biased by unknown and unquantified factors. It may be necessary to pretest and/or calibrate mixture ratios so that the curve is perceptually symmetric or of quantified asymmetry.

These data, combined with the overwhelming evidence of inhibition within odor mixtures at the receptor level (Araneda et al., 2000; Kay et al., 2003; Xu et al., 2020), make it clear that understanding mixture quality may not yet be a tractable theoretical problem at the level of neurophysiology. The best way forward for use of mixtures in neurophysiology is to test the psychophysical properties of each mixture to be used. The methods by which these mixtures are tested may be tuned to address specific qualitative questions. For example, pre-training animals to monomolecular mixture components and then asking them to identify a component favors overshadowing, as we have done here. Training animals to recognize a mixture and asking them to report whether any of the components or decoy odorants smell like the mixture favors discovery of configural or synthetic properties (Wiltrout et al., 2003; Kay et al., 2006; Frederick et al., 2009). In most cases, we might simply need to test each mixture individually. The specific odors and their combinations matter.

One limitation of our study is that odor detectability in binary mixtures in our study is used as a measure for odor intensity in the binary mixtures tested. Detectability could be influenced by olfactory intensity, trigeminal intensity and their interactions. Unlike testing human subjects, we cannot measure trigeminal intensity separately, by asking subjects to focus on different aspects of the odor experience. The goal of our study, however, is to examine the binary mixture percept as a whole, to ask rats which odor the mixture smells most like.

Our results align with human research in odor mixture quality. We show that binary mixture psychometric functions are not easily predictable from the components nor generalizable from mixture to mixture (Cometto-muñiz et al., 1989; Cometto-Muniz et al., 1999; Cometto-Muñiz et al., 2003a; Lindqvist et al., 2012). However, it should be noted that human and animal mixture perception studies are not directly comparable due to differences in odor delivery and perceptual testing protocols (Cometto-Muñiz et al., 2003b). In fact, few human studies of binary mixture perception have addressed trigeminality directly. Trigeminal profile may be one of the factors that give rise to odor quality, but odor quality is not directly measurable in animal research.

## Data availability statement

The raw data supporting the conclusions of this article will be made available by the authors, without undue reservation.

## Ethics statement

This animal study was reviewed and approved by the University of Chicago Institutional Animal Care and Use Committee.

## Author contributions

HL and LK designed the experiments and wrote the manuscript. HL constructed the behavioral apparatus, wrote the code for data collection, and analyzed the data. HL and CL trained the rats and collected data. All authors have approved the final version of the manuscript.
