# Supplementary material for: Testing effects of trigeminal stimulation on binary odor mixture quality in rats
Source: Front Neurosci. 2023 Mar 7;17:1059741. doi: 10.3389/fnins.2023.1059741 (PMC10027748; doi:10.3389/fnins.2023.1059741)
Supplement: Supplementary file 1 [file Data_Sheet_1.PDF]

Supplementary Material

Table S1

Bimodality test coefficients for all odor sets at all combination ratios. The criterion for significance in bimodality is > 0.555. The criterion for a bimodal overall distribution is >4 consecutive significant bimodal combination ratios. OS2(Eucalyptol/Limonene) and OS4 (Citral/PEA) have overall bimodal distributions.

	0	0.25	0.45	0.5	0.55	0.75	1
Eugenol/ PEA	0.4288	0.5314	0.4594	0.8089 *	0.5806 *	0.4449	0.5104
Eucalyptol/Limonene*	0.5427	0.6564 *	0.7781 *	0.8636 *	0.6533 *	0.7396 *	0.5873 *
Eugenol/ Cinnamaldehyde	0.4680	0.5011	0.4914	0.6589 *	0.5977 *	0.5866 *	0.4541
Citral/PEA*	0.4446	0.4759	0.7739 *	0.8239 *	0.7375 *	0.5637 *	0.6004 *

Figure S1.

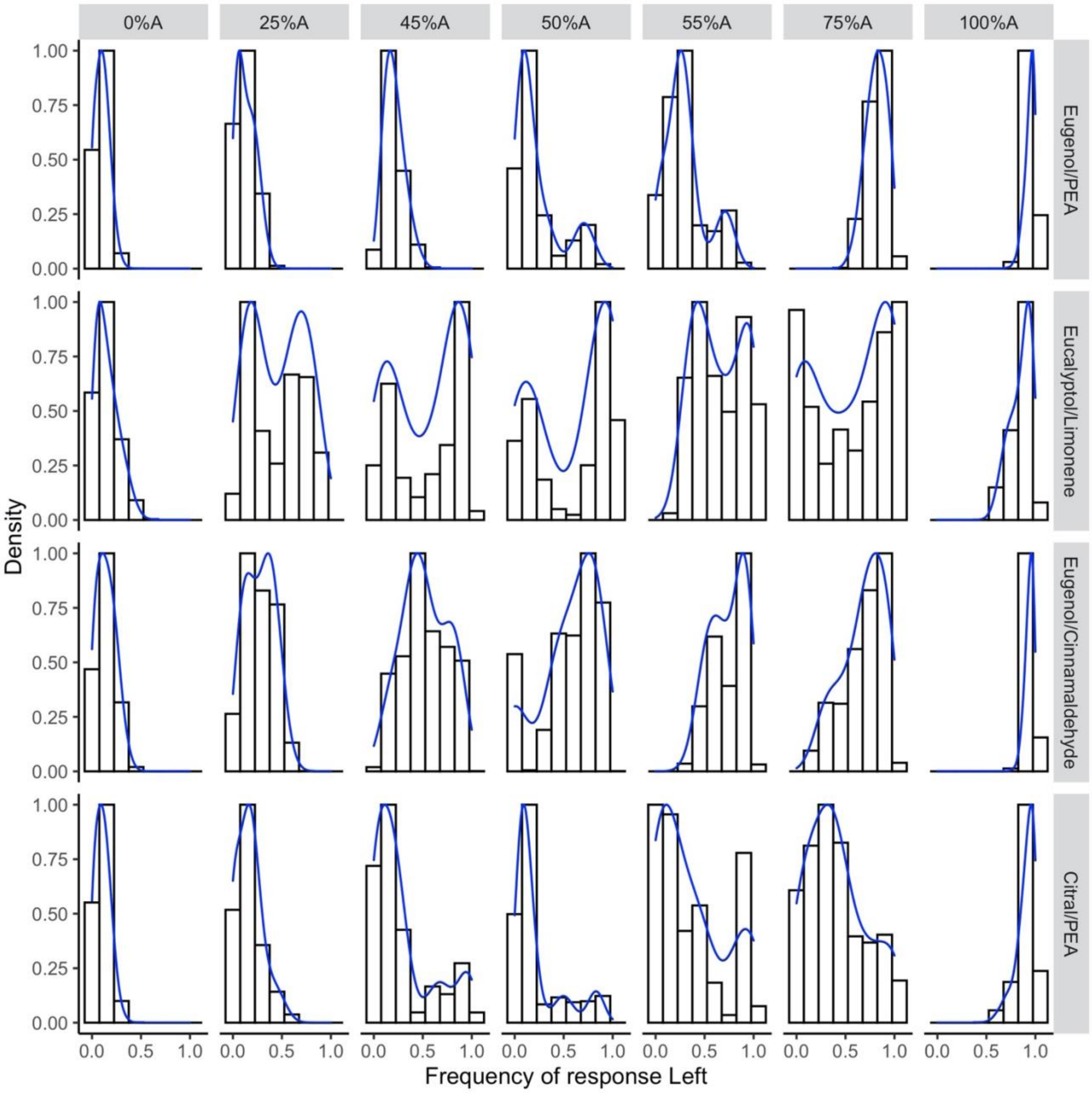

Supplementary Figure 1. Distributions of bootstrapped data.

Figure S2.

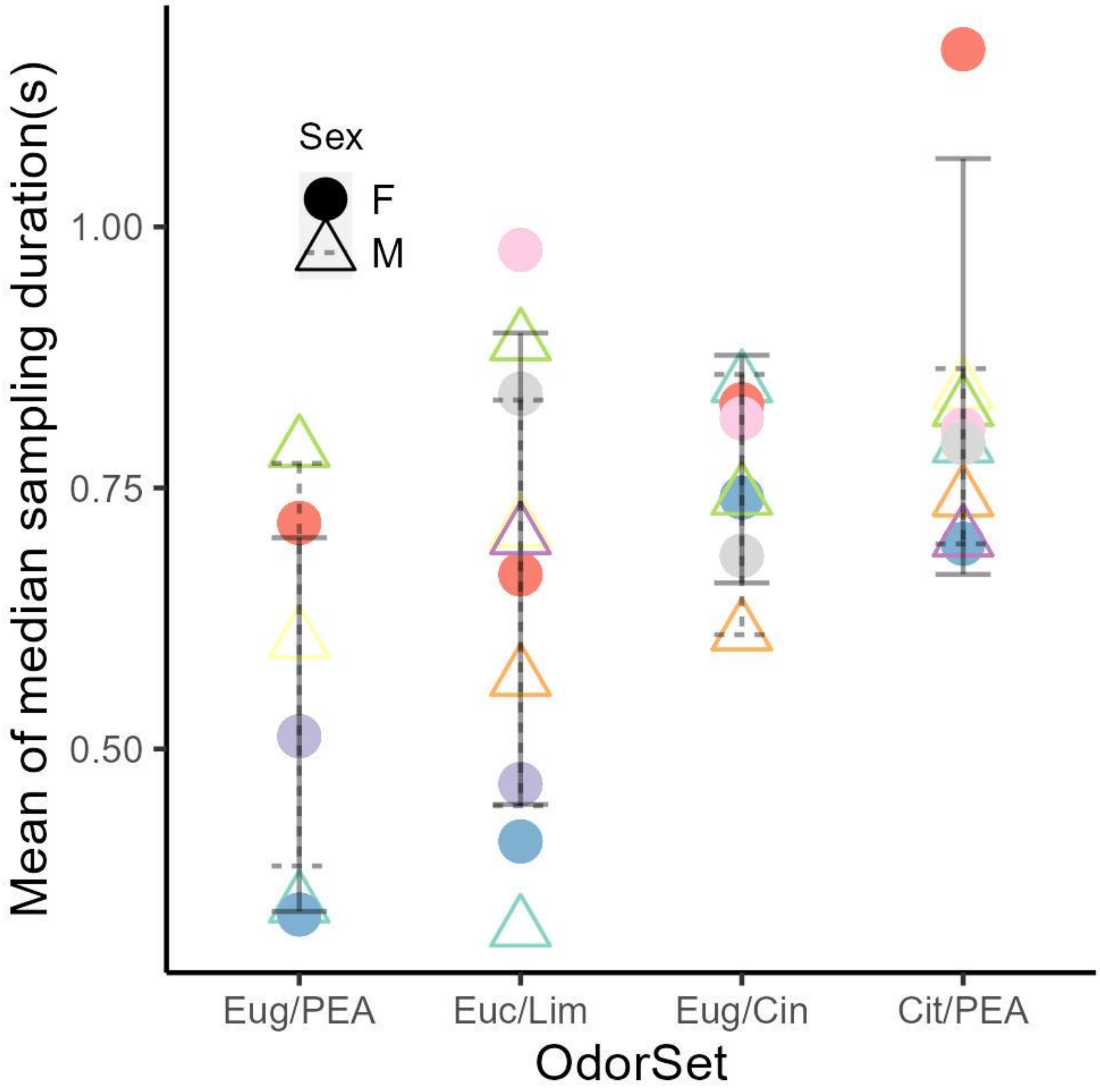

Supplementary Figure 2. Mean of median sampling duration by sex. Individual rats are identified by shape and color.
